# IgG anti-apolipoprotein A-1 antibodies in patients with systemic lupus erythematosus are associated with disease activity and corticosteroid therapy: an observational study

**DOI:** 10.1186/s13075-015-0539-z

**Published:** 2015-02-09

**Authors:** Sara Croca, Paul Bassett, Sharon Chambers, Maria Davari, Karim Fouad Alber, Oliver Leach, Yiannis Ioannou, Ian Giles, David Isenberg, Anisur Rahman

**Affiliations:** Centre for Rheumatology Research, University College London, 5 University Street, London, WC1E 6JF UK; Joint Research Office, UCL/University College London Hospital (UCLH)/Royal Free Hospital, Pond Street, London, NW3 2QG UK; Arthritis Research UK Centre for Adolescent Rheumatology (UCL/UCLH/Great Ormond Street Hospital), 5 University Street, London, WC1E 6JF UK

## Abstract

**Introduction:**

IgG anti-apolipoprotein A-1 (IgG anti-apoA-1) antibodies are present in patients with systemic lupus erythematosus (SLE) and may link inflammatory disease activity and the increased risk of developing atherosclerosis and cardiovascular disease (CVD) in these patients. We carried out a rigorous analysis of the associations between IgG anti-apoA-1 levels and disease activity, drug therapy, serology, damage, mortality and CVD events in a large British SLE cohort.

**Methods:**

Serum IgG anti-apoA-1 levels were measured in 100 healthy controls to define a cut-off for positivity. In 499 patients with SLE we obtained the earliest stored serum sample from their disease course and measured IgG anti-apoA-1 level. We then examined associations between IgG anti-apoA-1 positivity in early disease and the development of damage, CVD or death over a mean follow-up period of 12.1 years in these patients. In a separate study, we measured IgG anti-apoA-1 levels in 397 samples taken longitudinally from 49 patients with SLE over a mean period of 89 months of fluctuating disease activity and carried out multi-variable analysis to examine the demographic, serological, disease activity and treatment factors associated with IgG anti-apoA-1 level over time.

**Results:**

In the longitudinal study, IgG anti-apoA-1 levels were significantly higher in patients with persistently active disease, those on high dose corticosteroid and those not taking hydroxychloroquine. Of the 499 subjects who had early disease IgG anti-apoA-1 levels measured, 135 (27%) were positive. However, we found no convincing associations between early IgG anti-apoA-1 positivity and development of damage, mortality or CVD.

**Conclusions:**

IgG anti-apoA-1 developed early in a quarter of our patients with SLE, but this had no major impact on subsequent clinical outcomes. However, levels of IgG anti-apoA-1 vary over time and are associated with disease activity, treatment with high dose corticosteroid and not taking hydroxychloroquine.

## Introduction

Antibodies to apolipoprotein A-1 (anti-apoA-1) have been reported in the serum of patients with systemic lupus erythematosus (SLE) [1-4]. Several groups found independently that levels were elevated in patients with active SLE compared with inactive SLE [1-4], although none carried out multivariable analysis to exclude the effect of confounding factors. Apolipoprotein A-1 is the main constituent of high-density lipoprotein, which plays a major role in preventing atherosclerosis [5,6]. It has been suggested that anti-apoA-1 could interfere with this protective effect and thus promote atherosclerosis [2,4]. IgG anti-apoA-1 have also been associated with markers of increased plaque vulnerability such as intra-plaque macrophage, neutrophil and matrix metalloproteinase content [7] and are associated with increased levels of the surrogate plaque vulnerability markers interleukin-6, tumour necrosis factor alpha and matrix metalloproteinase-9 in patients with myocardial infarction [8]. Elevated IgG anti-apoA-1 levels are found in patients with acute coronary syndromes [9], are a risk factor for major cardiovascular events in the 12 months after myocardial infarction [10] and are associated with significantly increased risk of major cardiovascular events in patients with rheumatoid arthritis (RA) [11]. Anti-apoA-1 antibodies have thus attracted particular interest as a possible mediator between inflammation and the recognised increased risk of developing cardiovascular disease (CVD) in patients with SLE.

In a large multinational study of 9,547 patients with SLE there were 1,255 deaths, of which 313 were due to CVD [12]. Patients with SLE have fivefold to 10-fold greater risk of developing CVD than age-matched controls [13], rising to 50-fold in women between the ages of 35 and 44 [14]. Subclinical vascular disease is more common in patients with SLE than in age-matched and sex-matched controls [15,16]. Patients with SLE who suffer CVD events do so at a relatively young age – a mean of 49 years for women [13]. Standard methods based on the Framingham equations underestimate CVD risk in patients with SLE [17,18]. Alternative stratification tools to identify atherosclerosis – for example, vascular ultrasound – may be useful [19,20]. If IgG anti-apoA-1 have a true predictive value for CVD this could also be helpful. Previously, we did not find high IgG anti-apoA-1 levels in 24 patients with SLE who suffered CVD events [2]. Radwan and colleagues found no relationship between IgG anti-apoA-1 and carotid intima-media thickness in 80 Egyptian patients with SLE but, unlike most other SLE groups studied, none of their patients had carotid plaques [4].

Previous studies of IgG anti-apoA-1in patients with SLE were limited by having small numbers of patients, lack of multivariable analysis and short-term follow-up after the samples were taken. Here, we address these issues to answer the following questions: which demographic, serological, clinical and treatment factors are significantly associated with changing IgG anti-apoA-1 levels over time in patients with SLE? What is the prevalence of positivity for IgG anti-apoA-1 early in the disease course of SLE? Does positivity for IgG anti-apoA-1 early in the disease course predict subsequent damage, cardiovascular disease and/or mortality?

## Methods

Ethical approval for all parts of the study was granted by the joint University College London/University College London Hospitals Research Ethics Committee (Reference 06/Q0505/79) and subjects gave informed consent for use of their stored serum samples.

### Healthy control subjects

We tested samples from 100 healthy control subjects originally obtained as part of the Health Survey for England 2006 [21]. The samples were provided to us by the Health and Social Care Information Centre together with anonymised data on age, and the gender and ethnicity of the subjects and absence of long-term illness or previous CVD were confirmed. Their median age was 43 years (range 20 to 69), 49% were female and the ethnic distribution was 83% Caucasian, 8% Afro-Caribbean and 9% South Asian.

### Patients with systemic lupus erythematosus

The University College London Hospitals SLE clinic has been running since 1979 and we have followed over 600 patients with SLE since then, all fulfilling the revised American College of Rheumatology classification criteria [22]. From this population of patients we selected two different groups who were studied to answer different questions.

### Early disease group

For 499 patients we were able to obtain serum samples taken within 1 year of diagnosis and stored at −80°C. The earliest of these samples was obtained in 1978 and the most recent in 2011. We tested all of these samples for IgG anti-apoA-1 antibodies – using the enzyme-linked immunosorbent assay (ELISA) described below – to find out what proportion of patients with SLE have IgG anti-apoA-1 early in the disease course. These 499 patients with SLE had been under continuous follow-up for between 1 and 34 years (mean 12.1 years). We investigated whether positivity for IgG anti-apoA-1 within the first year of the disease was associated with the following outcomes: death from any cause; death before the age of 60; CVD – defined as either myocardial infarction with typical enzyme and/or ECG changes, ischaemic stroke confirmed by imaging or non-infarct coronary disease confirmed by angiography; and damage as measured by the Systemic Lupus International Collaborative Clinics Damage Index (SLICC-DI) [23].

Data on death and CVD were available for all 499 patients from review of their clinical records. Data on SLICC-DI scores were only available for 236 patients. These patients had been studied between 2006 and 2008 in a project designed specifically to collect comprehensive data on damage scores from patients who had been followed in the clinic for at least 10 years by then (that is, had been diagnosed between 1979 and 1996) [24]. Only 236 such patients were available and thus only SLICC-DI data from those patients are included in the current report.

For the early disease samples we did not have data on other autoantibodies, or on the disease activity or damage scores at the time of the sample.

### Longitudinal group

Longitudinal serum samples (*n* = 397) were selected retrospectively from a group of 49 patients with SLE with a mean of eight samples per patient (standard deviation (SD) 2.16; minimum 3; maximum 14) that had been obtained during a mean period of 89 months (SD 46; minimum 14; maximum 180) in the course of their disease. The patients were selected on the basis that they had varying levels of disease activity over time, including examples of flares in all of the main organs and systems of the body. By coincidence, 47 patients in this group were also members of the early disease group, but the longitudinal serum samples used in this part of the project were different from the early disease samples used in the other part of the project.

For all samples where data were available (94%), we obtained anti-dsDNA and complement C3 levels and disease activity from the date of the sample and from the previous three assessments. Anti-dsDNA and C3 were measured in the routine clinical laboratory at University College London Hospitals using the ELISA (Shield Diagnostics, Dundee, UK) and laser nephelometry respectively. Based on the normal limits for our laboratory, anti-dsDNA level >50 IU/ml was defined as high and C3 level <0.9 g/l as low.

Disease activity was measured using the classic British Isles Lupus Assessment Group (BILAG) index [25]. The more recent BILAG 2004 index was not used because many of the samples had been taken before 2004. Disease activity over the most recent four assessments was characterised as persistently low activity (all systems BILAG C, D or E) or persistently moderate–high activity (A or ≥1 B in any BILAG system on at least 2/4 occasions). Over 90% of all samples fell into one of those two categories and the rest were excluded from this part of the analysis. Global BILAG score was calculated using the formula A = 12, B = 5, C = 1, D = E = 0 as described previously [26].

Data on ethnicity, gender, drug therapy and the anti-Sm, anti-RNP, anti-Ro and anti-La (all tested by ELISA) status of the patients were obtained from the clinical records of the patients. We did not have data on SLICC-DI score at the time of each sample.

### Direct ELISA to detect IgG anti-ApoA-1 antibodies

IgG anti-ApoA-1 antibodies were detected by a modification of the direct ELISA protocol described previously [2,27]. All steps were carried out at 37°C except where specified. A Nunc-Maxisorb 96-well ELISA (Fisher Scientific, Loughborough, UK) plate was divided in half. One side (the test side) was coated with 10 μg/ml apolipoprotein A-1 (A0722; Sigma St Louis, Missouri, USA) in 70% ethanol. The other side (the control side) was coated with 70% ethanol. After incubation for 90 minutes, the plates were washed and blocked with 1% bovine serum albumin diluted in phosphate-buffered saline for 1 hour. Serum samples at 1:50 dilution in 1% bovine serum albumin–phosphate-buffered saline were tested in duplicate such that each sample was added to two test wells and two control wells. On each plate, a seven-point dilution of the positive control (a pool of six serum samples from patients known to have high serum IgG anti-apoA-1) was performed starting at 1:25 dilution. Following incubation for 1 hour, goat anti-human IgG–alkaline phosphatase conjugate (A3150; Sigma) diluted 1:1,000 in 1% bovine serum albumin–phosphate-buffered saline was added at room temperature for 1 hour followed by alkaline phosphatase substrate. Absorbance at 405 nm was recorded after 60 minutes. The net optical density (OD) reading for each sample was calculated by subtracting the OD in the control well from that in the matching test well to exclude nonspecific background binding. The mean net OD from the duplicate samples was converted to absorbance units (AU) by comparison with the standard curve of OD for the serial dilutions of the positive control sample on each plate. A value of 100 AU was defined as the OD given by a 1:50 dilution of the positive control sample. This assay was reproducible with intraplate and interplate coefficients of variation <10%.

### Statistical analysis

In the longitudinal group, assessment of anti-ApoA-1 levels showed a highly positive skewed distribution, which could not be transformed to a normally distributed scale. The outcomes were thus assumed to follow a negative binomial distribution. Owing to the longitudinal nature of this group, multiple samples for each patient were considered. To allow for the non-independence of the data, multilevel statistical methods were used for analysis. Two-level models were used with individual measurements clustered within patients. The analyses, performed using multilevel negative binomial regression, were performed in two stages. Firstly the separate effect of each factor upon the outcome was examined in a series of univariable analyses. Subsequently the joint effect of factors was examined in a multivariable analysis. A backward selection procedure was employed to retain only the statistically significant variables. Variance inflation factors were used to assess collinearity between variables, and as a result some variables that were collinear with other variables were excluded from the multivariable stage of the analysis.

In the early disease group, associations between anti-ApoA-1 levels and clinical outcomes were analysed using the statistical analysis software Prism. Univariable analysis was performed using the Mann–Whitney U test, as the sample did not follow a normal distribution. Statistical significance was considered when *P* <0.05. Survival curves for cardiovascular disease events and mortality were produced using the Kaplan–Meier method. For the analysis of mortality, patients who were not known to have died during the period of follow-up were censored at the end of the study period or at the time of loss to follow-up if that applied (for example, patients who moved away from London). For the analysis of CVD events, patients who had no such events were censored at the time of death, loss to follow-up or the end of the study period. The survival curves were compared using the log-rank test.

## Results

### Longitudinal group: elevated IgG anti-apoA-1 level was associated with high disease activity, high-dose steroids and not being treated with hydroxychloroquine

The mean age of the 49 patients in the longitudinal group was 36 years (SD 13.0) and 81% were female. Twenty-three patients were Caucasian, 18 were Afro-Caribbean and eight were other ethnicities. Twenty-one patients were anti-Ro-positive, six were anti-La-positive, 15 were anti-RNP-positive and 11 were anti-Sm-positive. During the follow-up period, 29 patients had at least one elevated anti-dsDNA level, 32 had at least one low C3 level and 46 suffered at least one flare (BILAG A or B in at least one system). Flares in all eight systems of the classic BILAG index were represented in the cohort.

**Figure 1:**
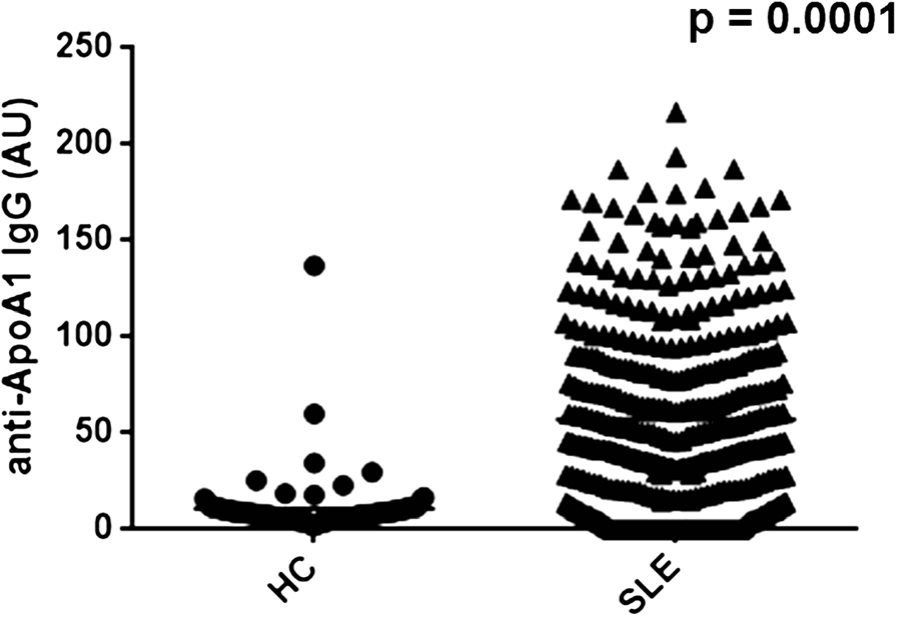
**Comparison of IgG anti-apoA-1 levels in serum samples from patients with SLE and healthy controls.** anti-apoA-1, antibodies to apolipoprotein A-1; AU, absorbance units; HC, healthy controls; IgG, immunoglobulin G; SLE, systemic lupus erythematosus.

Figure 1 shows that the IgG anti-apoA-1 level was higher in the 397 samples from patients with SLE (median 48.5, interquartile range (IQR) 16.0 to 87.5) than in the 100 healthy controls (median 8.0, IQR 5.9 to 10.7) (*P* = 0.0001). A positivity cutoff value was defined as the 97.5th percentile of 100 healthy controls (46.7 AU). Fifty per cent of the samples from patients with SLE were found to be IgG anti-ApoA-1-positive.

Table 1 presents the results of univariable analysis to assess association of 24 clinical, demographic and serological variables with serum IgG anti-apoA-1 level.

**Table 1:** **Univariable analysis of factors associated with serum IgG anti-apoA-1 levels in the longitudinal group**

**Variable**	**Category**	**Ratio (95% CI)**	***P*** **value**
Gender^a^	Female (*n* = 336)	1	
	Male (*n* = 61)	1.20 (0.85, 1.69)	0.30
Disease duration	Ratio given per 5-year increase	1.00 (0.90, 1.11)	0.99
Age (at diagnosis)	Ratio given per 10-year increase	1.02 (0.88, 1.18)	0.81
Ethnicity^a^	Caucasian (*n* = 182)	1	
	Afro-Caribbean (*n* = 146)	1.19 (0.90, 1.57)	
	Other (*n* = 69)	1.07 (0.75, 1.53)	0.46
Any ENA^b^	No (*n* = 165)	1	
	Yes (*n* = 232)	0.82 (0.63, 1.06)	0.13
Anti-Ro^b^	No (*n* = 226)	1	
	Yes (*n*-171)	0.90 (0.69, 1.17)	0.41
Anti-La^b^	No (*n* = 345)	1	
	Yes (*n* = 52)	0.54 (0.36, 0.80)	**0.002**
Anti-Sm^b^	No (*n* = 298)	1	
	Yes (*n* = 99)	0.64 (0.47, 0.87)	**0.004**
Anti-RNP^b^	No (*n* = 288)	1	
	Yes (*n* = 109)	1.11 (0.84, 1.47)	0.46
Anti-dsDNA level	<50 IU/ml (*n* = 177)	1	
	≥50 IU/ml (*n* = 170)	1.13 (0.90, 1.43)	0.29
C3 level	<0.9 g/l (*n* = 139)	1	
	≥0.9 g/l (*n* = 208)	0.82 (0.66, 1.03)	0.09
Disease activity in general system^c^	A, B (*n* = 31)	1	
	C, D, E (*n* = 344)	0.84 (0.60, 1.18)	0.32
Disease activity in mucocutaneous system^c^	A, B (*n* = 41)	1	
	C, D, E (*n* = 334)	0.89 (0.65, 1.22)	0.48
Disease activity in neuropsychiatric system^c^	A, B (*n* = 18)	1	
	C, D, E (*n* = 357)	0.93 (0.57, 1.52)	0.77
Disease activity in musculoskeletal system ^c^	A, B (*n* = 47)	1	
	C, D, E (*n* = 328)	0.80 (0.60, 1.08)	0.14
Disease activity in cardiorespiratory system^c^	A, B (*n* = 13)	1	
	C, D, E (*n* = 362)	0.66 (0.42, 1.02)	0.06
Disease activity in vascular system^c^	A, B (*n* = 12)	1	
	C, D, E (*n* = 363)	0.80 (0.47, 1.34)	0.40
Disease activity in renal system^c^	A, B (*n* = 41)	1	
	C, D, E (*n* = 329)	0.75 (0.55, 1.01)	0.06
Disease activity in haematological system^c^	A, B (*n* = 95)	1	
	C, D, E (*n* = 280)	0.63 (0.50, 0.79)	**<0.001**
Overall disease activity over last four assessments	Persistently low (*n* = 166)	1	
	Persistently moderate/high (*n* = 209)	1.30 (1.06, 1.59)	**0.01**
Hydroxychloroquine^d^	No (*n* = 223)	1	
	Yes (*n* = 174)	0.70 (0.55, 0.88)	**0.003**
Immunosuppression^d^	No (*n* = 200)	1	
	Yes (*n* = 197)	0.90 (0.73, 1.12)	0.35
Oral prednisolone^d^	≤7.5 mg/day (*n* = 101)	1	
	>7.5 mg/day (*n* = 296)	1.39 (1.13, 1.71)	**0.002**

Bold data indicate values of *P* <0.05. anti-apoA-1, antibodies to apolipoprotein A-1; BILAG, British Isles Lupus Assessment Group; CI, confidence interval; IgG, immunoglobulin G. ENA; antibodies to extractable nuclear antigens. ^a^For gender, *n* values refer to the numbers of samples taken from female and male subjects, rather than numbers of females and males in the cohort of patients. A similar stipulation applies to ethnicity, where *n* values refer to the number of samples taken from patients of each ethnic group. ^b^For ENA, anti-Ro, anti-La and anti-Sm, we did not have results from the date of every sample but it is assumed that positivity and negativity for these antigens generally remain stable. ^c^ ‘Disease activity in general system’ refers to the BILAG score (A, B, C, D or E) in the General Category of the BILAG index on the day when each sample was taken. The same principle applies to all of the other organ systems listed in the table, which are the eight different categories recorded in the BILAG. ^d^Refers to drugs being taken on the date of the sample.

IgG anti-apoA-1 levels were significantly lower in patients with positive anti-La and anti-Sm but there was no relationship with C3 or anti-dsDNA.

Patients with persistent moderate/high disease activity had 30% higher IgG anti-apoA-1 levels than those with persistently low activity. Those with haematological BILAG scores of A or B on the day of the sample had higher IgG anti-apoA-1 levels than those with scores of C, D or E (*P* <0.001). A similar trend was observed for patients with renal or cardiorespiratory A or B scores although statistical significance was not reached (*P* = 0.06 for both).

Patients treated with hydroxychloroquine had IgG anti-apoA-1 levels 30% lower than those who were not taking hydroxychloroquine, whereas those on higher dose prednisolone (>7.5 mg/day) had levels 39% higher than those taking lower doses.

Table 2 presents the results of multivariable analysis. No collinearity was observed between variables. Only negativity for anti-La and anti-Sm, haematological disease activity and taking higher dose prednisolone remained as independently associated variables.

**Table 2:** **Multivariable analysis of factors associated with IgG anti-apoA-1 level in the longitudinal cohort**

**Variable**	**Category**	**Ratio (95% CI)**	***P*** **value**
Anti-La	No	1	
	Yes	0.58 (0.39, 0.86)	0.006
Anti-Sm	No	1	
	Yes	0.58 (0.43, 0.80)	0.001
Activity in the haematological system	A, B	1	
	C, D, E	0.66 (0.53, 0.83)	<0.001
Steroids	≤7.5 mg/day	1	
	>7.5 mg/day	1.34 (1.08, 1.66)	0.008

anti-apoA-1, antibodies to apolipoprotein A-1; CI, confidence interval; IgG, immunoglobulin G.

**Figure 2:**
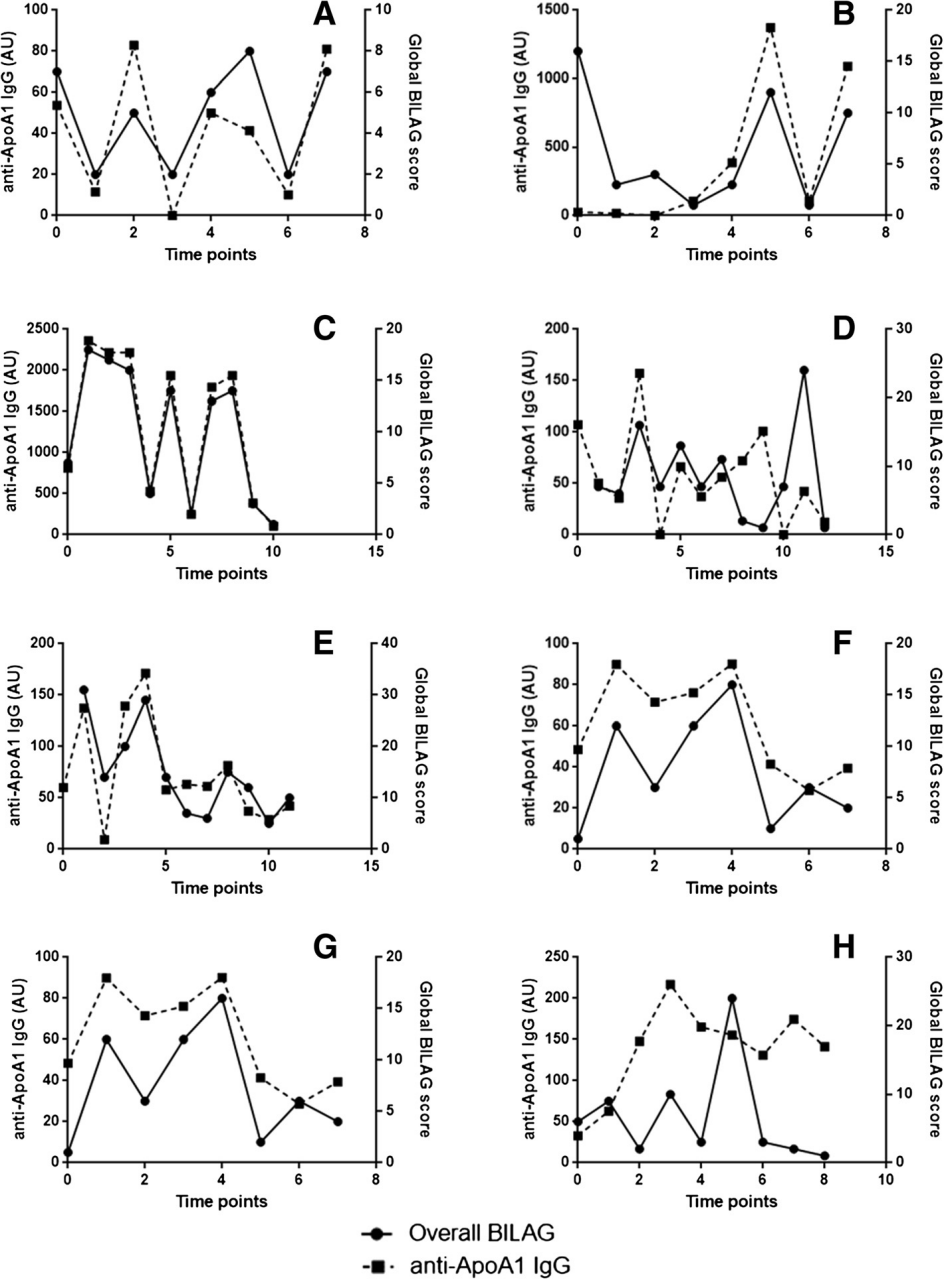
**Longitudinal variation in IgG anti-apoA-1 levels and global BILAG score in eight patients with SLE over time.** Time points on the *x* axis are successive follow-up visits. IgG anti-apoA-1 parallels global disease activity in many **(A)** to **(F)** but not all **(G),** (**H)** patients. anti-apoA-1, antibodies to apolipoprotein A-1; AU, absorbance units; BILAG, British Isles Lupus Assessment Group; IgG, immunoglobulin G.

As shown in Figure 2, IgG anti-apoA-1 levels varied over time and paralleled disease activity (measured by global BILAG score) closely in many (Figure 2A,B,C,D,E,F), but not all patients (Figure 2G,H).

### IgG anti-apoA-1 positivity was seen in 27% of early disease samples and was not associated with mortality or CVD

The mean age of the 499 subjects at the time when the early disease sample was taken was 30 years (SD 12.27, range 1 to 77) and 91.5% were female. Ethnic distribution was Caucasian 61%, African/Caribbean 20.5%, South Asian 11.4%, East Asian 4.6%, and other 2.5%.

Median IgG anti-apoA-1 levels were significantly higher in patients with SLE (median 21.4 AU, IQR 11.2 to 52.9) than in healthy controls (median 8.0 AU, IQR 5.9 to 10.7) (*P* <0.05) and 27% of patients tested positive in the first sample obtained after diagnosis of SLE. There was no correlation between the order of acquisition of samples and IgG anti-apoA-1 level (Spearman correlation coefficient −0.115), suggesting that storage time does not affect IgG anti-apoA-1 level.

As seen in the longitudinal group, there were no significant associations between IgG anti-apoA-1 level and age, gender or ethnicity.

**Figure 3:**
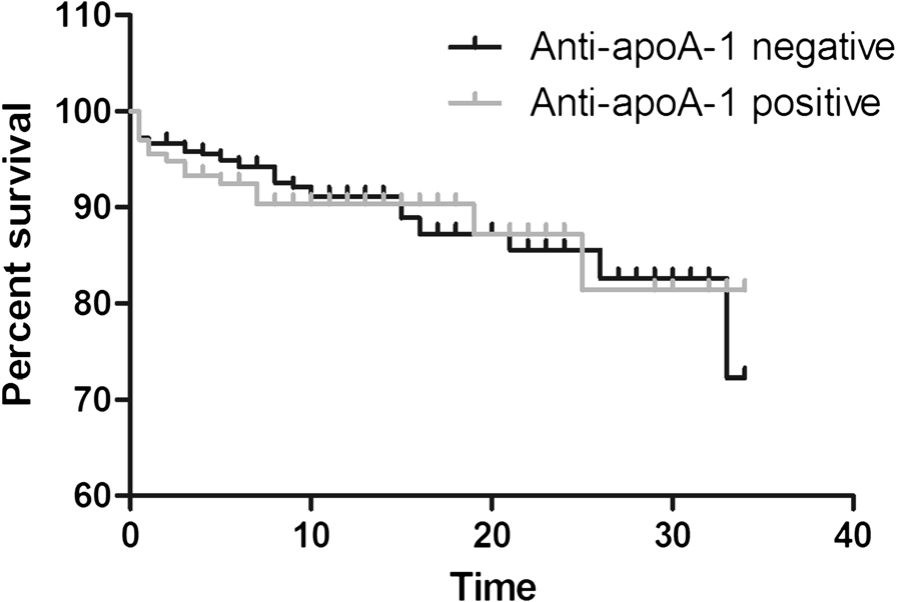
**Kaplan–Meier survival curve comparing percentage CVD-free survival in patients who were anti-apoA-1-positive (** ***n*** **= 135) and anti-apoA-1-negative (** ***n*** **= 364) in their early disease sample.** Time (*x* axis) measured in years. The number of subjects at risk at each time point was as follows: 499 at time 0, 275 at 10 years, 94 at 20 years and 27 at 30 years. anti-apoA-1, antibodies to apolipoprotein A-1; CVD, cardiovascular disease.

During follow-up, 40 patients (8.0%) suffered a CVD event; that is, coronary heart disease confirmed by enzyme or electrocardiography changes of myocardial infarction or by angiography for nonmyocardial infarction CAD or ischaemic stroke confirmed by imaging. The prevalence of CVD events did not differ between anti-apoA-1-positive and anti-apoA-1-negative patients (6.7% vs. 8.5%, *P* >0.05). A Kaplan–Meier survival curve showing the percentage of subjects in the anti-apoA-1-positive and anti-apoA-1-negative groups free of CVD at all time points up to 34 years of follow-up showed no significant difference between groups (Figure 3; *P* = 0.89 by log-rank test).

Similarly, positivity for IgG anti-apoA-1 did not predict onset of damage during follow-up. Table 3 presents damage scores at 5, 10, 15 and 20 years after stratification into quartiles based on the early disease sample IgG anti-apoA-1 level with no significant differences in damage score between the quartiles at any time point.

**Table 3:** **Mean damage scores over 20 years stratified by quartile of serum IgG anti-apoA-1 level in the early disease sample**

	**Lowest quartile**	**Second quartile**	**Third quartile**	**Highest quartile**
Damage score at 1 year (*n* = 236)	0.14	0.13	0.10	0.08
Damage score at 5 years (*n* = 236)	0.63	0.43	0.45	0.42
Damage score at 10 years (*n* = 209)	1.06	0.60	0.96	0.65
Damage score at 15 years (*n* = 131)	1.23	0.81	1.33	0.97
Damage score at 20 years (*n* = 69)	1.33	1.06	1.88	1.00

anti-apoA-1, antibodies to apolipoprotein A-1; IgG, immunoglobulin G.

During follow-up, 63.0% and 24.0% of patients were at some time positive for anti-dsDNA and anti-cardiolipin antibodies respectively. One-quarter of the patients were found to be rheumatoid factor-positive and over one-half were positive for antibodies to extractable nuclear antigens (14.9% anti-Sm, 27.8% anti-RNP, 37.5% anti-Ro and 13.9% anti-La).

Patients who developed anti-dsDNA positivity had higher IgG anti-apoA-1 in their early disease samples than those who did not (median 22.5 AU vs. 17.1 AU, *P* = 0.0012) and a similar relationship was found for those who developed anti-cardiolipin antibodies (IgG anti-apoA-1 25.5 AU vs. 21.0 AU, *P* = 0.025). There were no relationships with other autoantibodies.

**Figure 4:**
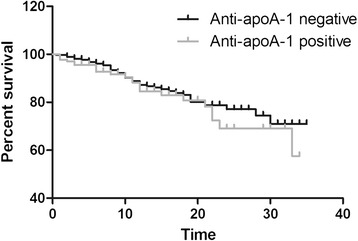
**Kaplan–Meier survival curve comparing percentage survival in patients who were anti-apoA-1-positive (** ***n*** **= 135) and anti-apoA-1-negative (** ***n*** **=364) in their early disease sample.** Time (*x* axis) measured in years. The number of subjects at risk at each time point was as follows: 499 at time 0, 290 at 10 years, 103 at 20 years and 31 at 30 years. anti-apoA-1, antibodies to apolipoprotein A-1.

Regarding mortality, 13% of patients died (*n* = 66), 48 of them before the age of 60. A Kaplan–Meier survival curve showing the percentage survival of subjects in the anti-apoA-1-positive and anti-apoA-1-negative groups at all time points up to 34 years of follow-up showed no significant difference between groups (Figure 4; *P* = 0.22 by log-rank test).

There was no indication that deaths in patients younger than 60 or in patients with raised IgG anti-apoA-1 were due disproportionately to CVD. In the over 60s, the causes of death were 17% CVD, 44% cancer, 17% infection, 6% renal and 17% other. In the under 60s, the causes were 13% CVD, 17% cancer, 31% infection, 8% renal and 31% other. Of the six patients who died from CVD under the age of 60, only one had positive IgG anti-apoA-1 at the time of diagnosis.

## Discussion

Previous studies confirmed the presence of IgG anti-apoA-1 antibodies in patients with SLE, indicated an association with higher disease activity and suggested a possible link with the development of CVD [1-4]. In this paper we have confirmed the association with disease activity by carrying out multivariable analysis of factors affecting IgG anti-apoA-1 levels in longitudinal samples from a group of 49 patients whose disease activity varied over time. We investigated the prevalence of elevated IgG anti-apoA-1 in early disease and its predictive value for CVD and mortality by studying 499 patients followed for a mean of 12.1 years after the date of the sample.

We showed that 27% of 499 patients were positive for IgG anti-apoA-1 very early in the disease course. Arbuckle and colleagues showed that many autoantibodies are present in patients with SLE even in stored samples taken before diagnosis of SLE [28]. However, anti-apoA-1 levels in early disease samples have not been studied before.

We found no association between IgG anti-apoA-1 positivity early in disease and increased risk of dying as survival curve analysis showed no difference between the anti-apoA-1-positive and anti-apoA-1-negative groups. Development of damage (due to disease activity or therapy) is a powerful risk factor for mortality [29] in SLE but we found no association between IgG anti-apoA-1 level and SLICC-DI score over a 10-year follow-up period in over 200 patients (Table 3).

We found no association between IgG anti-apoA-1 positivity early in disease and development of CVD later. The numbers of patients with CVD were relatively small (40/499). We have thus found no convincing link between IgG anti-apoA-1 and CVD in patients with SLE in this study. This finding agrees with those of Radwan and colleagues [4] in Egyptian patients with SLE but contrasts with those of Vuilleumier and colleagues, who found that, in 133 Swiss patients with RA followed for a median of 9 years [11], baseline IgG anti-apoA-1 positivity was strongly associated with risk of developing a major cardiovascular event (hazard ratio 4.7, 95% confidence interval 1.9 to 11.2), even after adjusting for standard CVD risk factors in multivariable analysis. However, this RA cohort was older (mean age 65 years), contained more males (30%) and had higher rate of CVD events compared with our group or other SLE groups reported in the literature [1,4]. Longer-term studies may well be needed in SLE than in RA to identify predictive effects of factors such as autoantibodies on CVD risk. Notably, in a large inception cohort study, 1,249 patients with SLE were followed for a median of 8 years and only older age and male gender were identified as CVD risk factors in multivariable analysis [30].

Our study had a number of limitations. We did not study different isotypes of IgG anti-apoA-1 antibodies. In future, it may be worthwhile to investigate whether specific isotypes are more strongly associated with overall or organ-specific disease activity. We defined CVD by review of the notes and did not have detailed information on CVD events (such as troponin rise and degree of arterial stenosis). Other studies, such as those of Vuilleumier and colleagues defined CVD using harder endpoints [9,11]. The number of patients in the longitudinal group was relatively small at 49. The surprising finding that elevated IgG anti-apoA-1 levels were associated with negativity for anti-Sm and anti-La thus needs to be confirmed in a larger cohort. If this is confirmed, it may be useful to investigate whether associations of IgG anti-apoA-1 and different forms of disease activity are different in anti-Sm-positive or anti-La-positive versus anti-Sm-negative or anti-La-negative patients.

Previously we showed that IgG anti-apoA-1 levels were higher in patients with persistently active disease than quiescent disease and rose at times of disease flare [2]. However, we did not study serial samples taken over long periods and were unable to carry out multivariable analysis. By studying samples taken from 49 patients longitudinally, we have confirmed the association of IgG anti-apoA-1 level with disease activity, particularly haematological activity. The association with haematological activity, however, may arise partly from the fact that there were more patients with BILAG A or B scores in the haematology system than in any other system. In previous papers we have also demonstrated associations between serological markers and activity in particular systems: anti-nucleosome antibodies and renal lupus [31], interleukin-6 and haematological lupus [32], and nitrated nucleosomes and vasculitis [33]. Hydroxychloroquine was associated with lower IgG anti-apoA-1, and high-dose corticosteroids with higher anti-apoA-1. In many patients, IgG anti-apoA-1 levels varied in parallel with disease activity over time. These results, however, do not prove a causal relationship between presence of IgG anti-apoA-1 antibodies and development of inflammation or disease activity in SLE. The possibility of a causal relationship would be an interesting question for future work.

## Conclusion

In summary, this comprehensive analysis shows that IgG anti-apoA-1 antibodies are found commonly in patients with SLE and develop early in the disease course. Levels are associated with high disease activity and treatment with corticosteroids, but we have shown no convincing link with CVD. It remains possible that such a link will be shown in longer-term studies or by studying more sensitive measures of atherosclerosis such as plaque echogenicity, thickness, area or volume using vascular ultrasound scanning [34,35].

## Abbreviations

anti-apoA-1: antibodies to apolipoprotein A-1
AU: absorbance units
BILAG: British Isles Lupus Assessment Group
CVD: cardiovascular disease
ELISA: enzyme-linked immunosorbent assay
IgG: immunoglobulin G
IQR: interquartile range
OD: optical density
RA: rheumatoid arthritis
SD: standard deviation
SLE: systemic lupus erythematosus
SLICC-DI: Systemic Lupus International Collaborative Clinics Damage Index

## References

[1] Batuca JR, Ames PR, Amaral M, Favas C, Isenberg DA, Delgado AJ (2009). Anti-atherogenic and anti-inflammatory properties of high-density lipoprotein are affected by specific antibodies in systemic lupus erythematosus. Rheumatology (Oxford).

[2] O’Neill SG, Giles I, Lambrianides A, Manson J, D’Cruz D, Schrieber L (2010). Antibodies to apolipoprotein A-I, high-density lipoprotein, and C-reactive protein are associated with disease activity in patients with systemic lupus erythematosus. Arthritis Rheum.

[3] Shoenfeld Y, Szyper-Kravitz M, Witte T, Doria A, Tsutsumi A, Tatsuya A (2007). Autoantibodies against protective molecules – C1q, C-reactive protein, serum amyloid P, mannose-binding lectin, and apolipoprotein A1: prevalence in systemic lupus erythematosus. Ann N Y Acad Sci.

[4] Radwan MM, El-Lebedy D, Fouda R, Elsorougy E, Fakhry D (2014). Anti-apoliprotein A-1 antibodies and carotid intima-media thickness in Egyptian women with systemic lupus erythematosus. Clin Rheumatol.

[5] Bruce IN (2005). ‘Not only … but also’: factors that contribute to accelerated atherosclerosis and premature coronary heart disease in systemic lupus erythematosus. Rheumatology (Oxford).

[6] Hahn BH, Grossman J, Chen W, McMahon M (2007). The pathogenesis of atherosclerosis in autoimmune rheumatic diseases: roles of inflammation and dyslipidemia. J Autoimmun.

[7] Montecucco F, Vuilleumier N, Pagano S, Lenglet S, Bertolotto M, Braunersreuther V (2011). Anti-Apolipoprotein A-1 auto-antibodies are active mediators of atherosclerotic plaque vulnerability. Eur Heart J.

[8] Pagano S, Satta N, Werling D, Offord V, de Moerloose P, Charbonney E (2012). Anti-apolipoprotein A-1 IgG in patients with myocardial infarction promotes inflammation through TLR2/CD14 complex. J Intern Med.

[9] Vuilleumier N, Reber G, James R, Burger D, de Moerloose P, Dayer JM (2004). Presence of autoantibodies to apolipoprotein A-1 in patients with acute coronary syndrome further links autoimmunity to cardiovascular disease. J Autoimmun.

[10] Vuilleumier N, Rossier MF, Pagano S, Python M, Charbonney E, Nkoulou R (2010). Anti-apolipoprotein A-1 IgG as an independent cardiovascular prognostic marker affecting basal heart rate in myocardial infarction. Eur Heart J.

[11] Vuilleumier N, Bas S, Pagano S, Montecucco F, Guerne PA, Finckh A (2010). Anti-apolipoprotein A-1 IgG predicts major cardiovascular events in patients with rheumatoid arthritis. Arthritis Rheum.

[12] Bernatsky S, Boivin JF, Joseph L, Manzi S, Ginzler E, Gladman DD (2006). Mortality in systemic lupus erythematosus. Arthritis Rheum.

[13] Elliott JR, Manzi S, Edmundowicz D (2007). The role of preventive cardiology in systemic lupus erythematosus. Curr Rheumatol Rep.

[14] Manzi S, Meilahn EN, Rairie JE, Conte CG, Medsger TA, Jansen-McWilliams L (1997). Age-specific incidence rates of myocardial infarction and angina in women with systemic lupus erythematosus: comparison with the Framingham Study. Am J Epidemiol.

[15] Roman MJ, Shanker BA, Davis A, Lockshin MD, Sammaritano L, Simantov R (2003). Prevalence and correlates of accelerated atherosclerosis in systemic lupus erythematosus. N Engl J Med.

[16] Asanuma Y, Oeser A, Shintani AK, Turner E, Olsen N, Fazio S (2003). Premature coronary-artery atherosclerosis in systemic lupus erythematosus. N Engl J Med.

[17] Esdaile JM, Abrahamowicz M, Grodzicky T, Li Y, Panaritis C, du Berger R (2001). Traditional Framingham risk factors fail to fully account for accelerated atherosclerosis in systemic lupus erythematosus. Arthritis Rheum.

[18] Bessant R, Hingorani A, Patel L, MacGregor A, Isenberg DA, Rahman A (2004). Risk of coronary heart disease and stroke in a large British cohort of patients with systemic lupus erythematosus. Rheumatology (Oxford).

[19] Thompson T, Sutton-Tyrrell K, Wildman RP, Kao A, Fitzgerald SG, Shook B (2008). Progression of carotid intima-media thickness and plaque in women with systemic lupus erythematosus. Arthritis Rheum.

[20] Elliott JR, Manzi S, Sattar A, Santelices LC, Avram Z, Shaw P (2008). Carotid intima-media thickness and plaque predict future cardiovascular events in women with systemic lupus erythematosus. Arthritis Rheum.

[21] Craig R, Mindell J (2008). Health Survey for England – 2006, CVD and risk factors for adults, obesity and risk factors for children.

[22] Hochberg MC (1997). Updating the American College of Rheumatology revised criteria for the classification of systemic lupus erythematosus. Arthritis Rheum.

[23] Gladman DD, Goldsmith CH, Urowitz MB, Bacon P, Fortin P, Ginzler E (2000). The Systemic Lupus International Collaborating Clinics/American College of Rheumatology (SLICC/ACR) Damage Index for Systemic Lupus Erythematosus International Comparison. J Rheumatol.

[24] Chambers SA, Allen E, Rahman A, Isenberg D (2009). Damage and mortality in a group of British patients with systemic lupus erythematosus followed up for over 10 years. Rheumatology (Oxford).

[25] Hay EM, Bacon PA, Gordon C, Isenberg DA, Maddison P, Snaith ML (1993). The BILAG index: a reliable and valid instrument for measuring clinical disease activity in systemic lupus erythematosus. Q J Med.

[26] Cresswell L, Yee CS, Farewell V, Rahman A, Teh LS, Griffiths B (2009). Numerical scoring for the Classic BILAG index. Rheumatology (Oxford).

[27] Delgado Alves J, Kumar S, Isenberg DA (2003). Cross-reactivity between anti-cardiolipin, anti-high-density lipoprotein and anti-apolipoprotein A-I IgG antibodies in patients with systemic lupus erythematosus and primary antiphospholipid syndrome. Rheumatology (Oxford).

[28] Arbuckle MR, McClain MT, Rubertone MV, Scofield RH, Dennis GJ, James JA (2003). Development of autoantibodies before the clinical onset of systemic lupus erythematosus. N Engl J Med.

[29] Rahman P, Gladman DD, Urowitz MB, Hallett D, Tam LS (2001). Early damage as measured by the SLICC/ACR damage index is a predictor of mortality in systemic lupus erythematosus. Lupus.

[30] Urowitz MB, Gladman D, Ibanez D, Bae SC, Sanchez-Guerrero J, Gordon C (2010). Atherosclerotic vascular events in a multinational inception cohort of systemic lupus erythematosus. Arthritis Care Res.

[31] Manson JJ, Ma A, Rogers P, Mason LJ, Berden JH, van der Vlag J (2009). Relationship between anti-dsDNA, anti-nucleosome and anti-alpha-actinin antibodies and markers of renal disease in patients with lupus nephritis: a prospective longitudinal study. Arthritis Res Ther.

[32] Ripley BJ, Goncalves B, Isenberg DA, Latchman DS, Rahman A (2005). Raised levels of interleukin 6 in systemic lupus erythematosus correlate with anaemia. Ann Rheum Dis.

[33] Croca S, Bassett P, Pericleous C, Alber KF, Latchman D, Isenberg D (2014). Serum nitrated nucleosome levels in patients with systemic lupus erythematosus: a retrospective longitudinal cohort study. Arthritis Res Ther.

[34] Johnsen SH, Mathiesen EB, Joakimsen O, Stensland E, Wilsgaard T, Lochen ML (2007). Carotid atherosclerosis is a stronger predictor of myocardial infarction in women than in men: a 6-year follow-up study of 6226 persons: the Tromso Study. Stroke.

[35] Mallett C, House AA, Spence JD, Fenster A, Parraga G (2009). Longitudinal ultrasound evaluation of carotid atherosclerosis in one, two and three dimensions. Ultrasound Med Biol.

